# Biology of *Francisella tularensis* Subspecies *holarctica* Live Vaccine Strain in the Tick Vector *Dermacentor variabilis*

**DOI:** 10.1371/journal.pone.0035441

**Published:** 2012-04-18

**Authors:** Rinosh J. Mani, Mason V. Reichard, Rebecca J. Morton, Katherine M. Kocan, Kenneth D. Clinkenbeard

**Affiliations:** Department of Veterinary Pathobiology, Center for Veterinary Health Sciences, Oklahoma State University, Stillwater, Oklahoma, United States of America; University of Louisville, United States of America

## Abstract

**Background:**

The γ-proteobacterium *Francisella tularensis* is the etiologic agent of seasonal tick-transmitted tularemia epizootics in rodents and rabbits and of incidental infections in humans. The biology of *F. tularensis* in its tick vectors has not been fully described, particularly with respect to its quanta and duration of colonization, tissue dissemination, and transovarial transmission. A systematic study of the colonization of *Dermacentor variabilis* by the *F. tularensis* subsp. *holarctica* live vaccine strain (LVS) was undertaken to better understand whether *D. variabilis* may serve as an inter-epizootic reservoir for *F. tularensis*.

**Methodology/Principal Findings:**

Colony-reared larva, nymph, and adult *D. variabilis* were artificially fed LVS via glass capillary tubes fitted over the tick mouthparts, and the level of colonization determined by microbial culture. Larvae and nymphs were initially colonized with 8.8±0.8×10^1^ and 1.1±0.03×10^3^ CFU/tick, respectively. Post-molting, a significant increase in colonization of both molted nymphs and adults occurred, and LVS persisted in 42% of molted adult ticks at 126 days post-capillary tube feeding. In adult ticks, LVS initially colonized the gut, disseminated to hemolymph and salivary glands by 21 days, and persisted up to 165 days. LVS was detected in the salivary secretions of adult ticks after four days post intra-hemocoelic inoculation, and LVS recovered from salivary gland was infectious to mice with an infectious dose 50% of 3 CFU. LVS in gravid female ticks colonized via the intra-hemocoelic route disseminated to the ovaries and then to the oocytes, but the pathogen was not recovered from the subsequently-hatched larvae.

**Conclusions/Significance:**

This study demonstrates that *D. variabilis* can be efficiently colonized with *F. tularensis* using artificial methods. The persistence of *F. tularensis* in *D. variabilis* suggests that this tick species may be involved in the maintenance of enzootic foci of tularemia in the central United States.

## Introduction

*Francisella tularensis* is a highly infectious, Gram-negative, coccobacillus that causes tularemia epizootics in small mammals and incidental infections in humans [1], [2], [3]. Although *F. tularensis* can infect a wide range of animal hosts, including reptiles and birds, maintenance of the agent in an endemic region involves small mammalian hosts which maintain a tick or biting-insect parasitic cycle in which the arthropods serve as vectors of *F. tularensis* [3], [4]. Ticks have been implicated as the primary vector for *F. tularensis* in many endemic regions, but biting flies and mosquitoes are also reported to serve as primary vectors. In contrast to biting flies and mosquitoes which appear to serve as only mechanical vectors, ticks serve as biological vectors with the bacterium colonizing, multiplying and persisting in the vector [4], [5]. Ticks have co-evolved with *Francisella* species as demonstrated by the presence of *Francisella*-like endosymbionts in many tick species [6], [7], [8], [9]. Although ticks and their hosts are reported to maintain tularemia epizootics in nature, the biology of *F. tularensis* in ticks has not been fully characterized.

Rodents and lagomorphs are thought to serve as primary hosts, and their associated tick species serve as vectors in the endemic tularemia foci of the central United States in Arkansas, Missouri, and Oklahoma [10], [11], [12]. In Oklahoma, human tularemia occurs in a summer seasonal pattern likely mirroring the seasonality of tularemia in small mammals. Peaks of this seasonality are coincident with questing activity of adult *Dermacentor variabilis* and nymphal and adult *Amblyomma americanum*. These tick species are thought to be the primary vectors for human tularemia in this region [11], [13], [14]. The cross-timber and prairie-forest ecosystems in this hyperendemic region are especially suitable for these ticks, which are reported to be involved in 60–70% of human tularemia cases in this area [11], [12]. Both *D. variabilis* and *A. americanum* have been shown to be experimental vectors for *F. tularensis*, and transstadial transmission from larva to nymph and nymph to adult has been reported [15], [16]. Although *Francisella*-like endosymbionts are transmitted transovarially in ticks, studies of transovarial transmission of *F. tularensis* in ticks have reported conflicting results [15], [16], [17].

Detailed systematic studies regarding duration and quanta of colonization, tissue localization, and transovarial transmission of *F. tularensis* in tick vectors have not been reported. For the present study artificial glass capillary tube feeding was used to colonize *D. variabilis* with *F. tularensis* subspecies *holarctica* strain LVS. This method of introduction of *F. tularensis* into ticks allows convenient exposure of large numbers of ticks to a known and reproducible level of bacteria in the inocula thereby avoiding the variability of inocula size, uncertainty of synchronizing tick feeding with bacteremia, and the mortality of infected animals used for colonization of ticks with *F. tularensis* by acquisition tick feeding [15], [18]–[20].

This study is the first report of the use of artificial methods for the colonization of tick vectors with *F. tularensis*. We found that LVS colonization persisted in *D. variabilis* nymphs and adults for extended periods, but nadirs of colonization quanta occurred prior to transstadial transmission. In adult ticks, LVS multiplied and disseminated from gut to salivary glands from which it was infectious for mice, and to ovaries, but LVS was not transovarially transmitted. Based on these observations, adult *D. variabilis* have the potential to serve as inter-epizootic reservoirs for persistence of tularemia in the central United States.

## Results

### LVS colonization of *D. variabilis* larvae and transstadial transmission to nymphs

**Figure 1:**
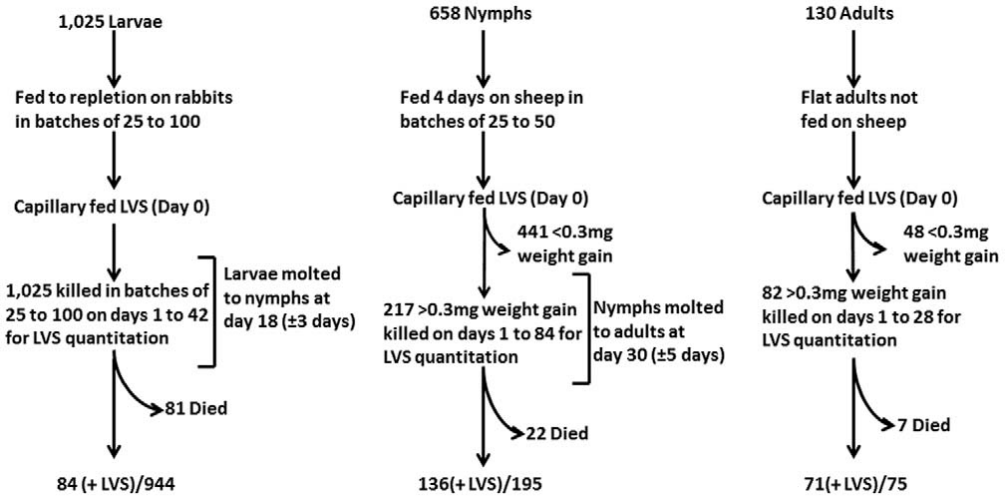
Quantum and duration of colonization and transstadial transmission of LVS experimental design. Larvae, nymphs and adults *D. variabilis* in batches of various sizes were capillary fed tick meal containing LVS. Ingestion of capillary fed tick meal was determined for nymph and adult ticks by post-feeding weight gains of ≥0.3 mg. Nymphs and adults showing <0.3 mg weight gains were excluded from further analysis. The overall rate of ticks positive for LVS for each tick life cycle stage capillary tube fed LVS is given as total number of ticks which were positive for LVS (+LVS)/total number of ticks tested.

**Figure 2:**
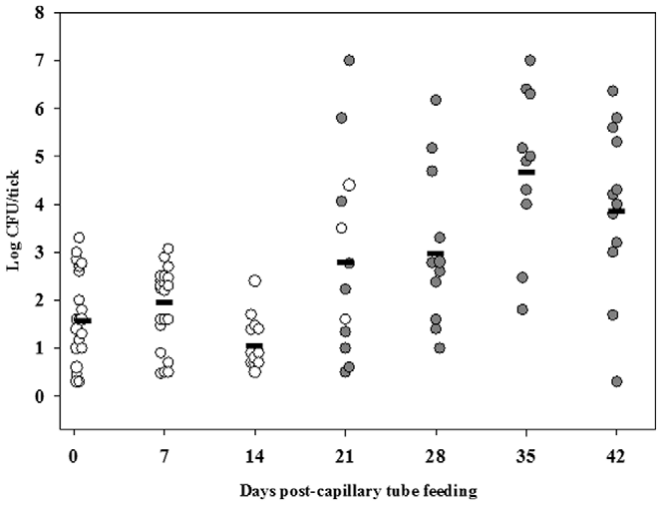
LVS is transmitted transstadially from larvae to nymphs. Open circles are capillary tube fed larvae and filled circles are molted nymphs. The calculated mean CFU/tick for colonized larvae and molted nymphs for each time point is represented by the horizontal line. For each time point, n was 10.

To assess quanta and duration of colonization and transstadial transmission of *F. tularensis* in larval *D. variabilis*, larvae fed to repletion on rabbits were exposed to LVS by capillary feeding tick meal containing 10^7^ CFU/mL LVS. On days one through forty-two post-capillary feeding, groups of larvae were assayed for LVS (Fig. 1). At one day post-capillary tube feeding, 10 of 53 (18.6%) of larvae tested were positive by microbial culture for LVS with a mean colonization level of 8.8±0.8×10^1^ CFU/larva (Fig. 2; limit of detection was 2 CFU/larva). The low percent of larvae which were colonized with LVS via capillary feeding is likely from a lack of ingestion of tick meal because the larvae had been fed to repletion on rabbits prior to capillary feeding. Larvae molted to nymphs at 18 days post-capillary tube feeding. The level of colonization declined to 1.2±0.25×10^1^ CFU/pre-molted larvae around the time of molting, but post-molting the mean colonization level increased to 5.2±0.01×10^4^ CFU/molted nymph at 35 days post-capillary tube feeding, confirming larval to nymph transstadial passage of LVS. A significant increase in the mean colonization levels between 14 days and 35 days post-capillary tube feeding (unadjusted *P*<0.001) was observed demonstrating that LVS multiplication occurred in ticks after molting.

### LVS colonization of *D. variabilis* nymphs and transstadial development in the subsequently molted adults

**Figure 3:**
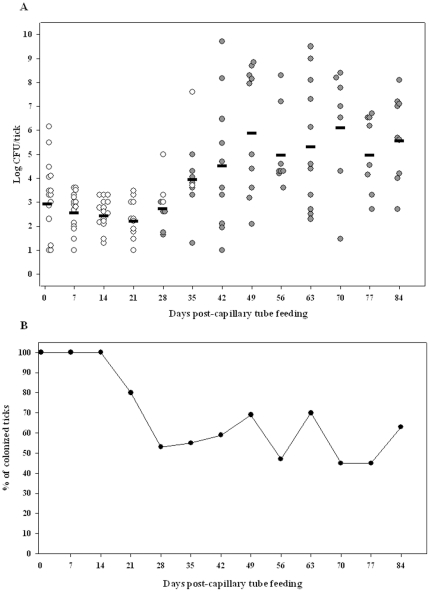
LVS is transmitted transstadially from nymphs to adults. (A) Open circles are capillary tube fed nymphs and filled circles are molted adults. The calculated mean CFU/tick for colonized nymphs and molted adults for each time point is represented by the horizontal line. For each time point up to day 84, n was 15. (B) Percentage of colonized ticks in the same experiment.

To assess quanta and duration of colonization and transstadial transmission of *F. tularensis* in *D. variabilis* nymphs, nymphs fed for 4 days on sheep were exposed to LVS by capillary feeding tick meal containing 10^7^ CFU/mL LVS. Approximately 30% of capillary tube fed-nymphs ingested tick meal as evidenced by weight gains of >0.3 mg during capillary tube feeding and were used for assessment of LVS colonization (Fig. 1). On days one through eighty-four post-capillary feeding, groups of nymphs were assayed for LVS (Fig. 3). These colonized nymphs had a mean level of 1.1±0.03×10^3^ CFU/nymph at one day post-capillary tube feeding with 100% remaining colonized for 14 days (n = 15). However, the percent colonization declined towards molting to adults at 28 days, at which time 47% were negative for LVS by culture indicating that either the colonization was too low to be detected by culture (<10 CFU/tick) and RT-qPCR (<5 CFU/tick) or these nymphs may have cleared colonization (Fig. 3). Interestingly, 78% of nymphs molting at 28 days had cleared LVS colonization and only 25% of colonized nymphs molted by 28 days, suggesting that colonization may prolong the time to molting. Following molting to adults, the percent of colonized molted adults remained relatively constant at 60% for post-capillary tube feeding days 42 to 84. The mean colonization level was 2.0±0.06×10^2^ at post-capillary tube feeding day 21 and increased to 1.0±0.001×10^6^ CFU/molted adult at day 49 post-capillary tube feeding. Among the molted adults, 60% female ticks were colonized whereas 49% of male ticks were colonized with LVS (not statistically significant). Both sexes had a similar mean colonization level of 7.9×10^4^ CFU/molted adult. Forty-two percent of adults colonized with LVS as capillary fed nymphs maintained colonization through 126 days (longest time point of observation).

### LVS colonization of *D. variabilis* adults

**Figure 4:**
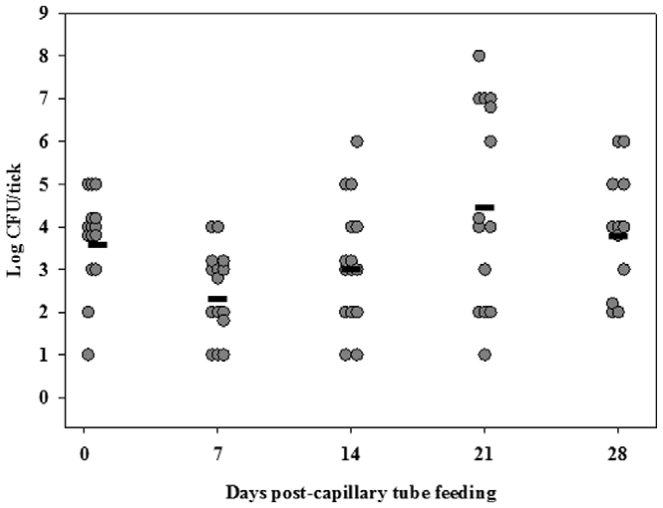
LVS colonization of adult *D. variabilis*. The filled circles are capillary tube fed adults. The calculated mean CFU/tick for colonized unfed adults for each time point is represented by the horizontal line. For each time point, n was 15.

To assess quanta and duration of colonization of *F. tularensis* in *D. variabilis* adults, flat adults which had not been fed a blood meal as adults were exposed to LVS by capillary feeding tick meal containing 10^7^ CFU/mL LVS. Fifty-seven percent of capillary tube fed flat adults ingested tick meal as evidenced by weight gains of >0.3 mg during capillary tube feeding and were used for assessment of LVS colonization (Fig. 1). On days one through twenty-eight post-capillary feeding, groups of adults were assayed for LVS (Fig. 4). A range of mean LVS colonization levels of 10^2^ to 10^4^ CFU/tick for the 28 days post-capillary tube feeding period was observed. Twenty-five percent of flat adults colonized with LVS as capillary fed flat adults maintained colonization through 165 days (longest time point of observation).

### Determination of inoculum dose of LVS necessary to establish colonization of *D. variabilis* adults

To determine the minimum number of *F. tularensis* necessary to establish colonization of adults ticks, flat adults were exposed to LVS via fine needle injection of the agent into the body cavity of the tick. As shown in Table 1, an inoculum dose of only 1.5 CFU/tick delivered by intra-hemocoelic (i.h.) injection was sufficient to establish colonization in 38% of ticks by day 14 post-injection compared to a high inoculation dose (140 CFU/tick) which established colonization in 100% of the ticks at one day post injection. The mean level of colonization for ticks inoculated with 1.5 CFU/tick at 14 days post injection was 5.0±2.2×10^1^ CFU/tick. At low doses, there appears to be a lag between inoculation and sufficient colonization to reach the limit of detection of the colonization assay (2 CFU/tick).

**Table 1:** Determination of dose of LVS necessary to establish colonization of adult *D. variabilis* ticks.

Days post injection	% Colonized per inoculum dose		
	1.5 CFU/tick	12.5 CFU/tick	140 CFU/tick
Day 1	0%†	40%§	100%§
Day 7	0%†	80%§	100%§
Day 14	38%‡	ND*	ND*

† n = 5,

‡ n = 8,

§ n = 10,

* data not collected.

### Tissue localization of LVS in *D. variabilis* adults

**Figure 5:**
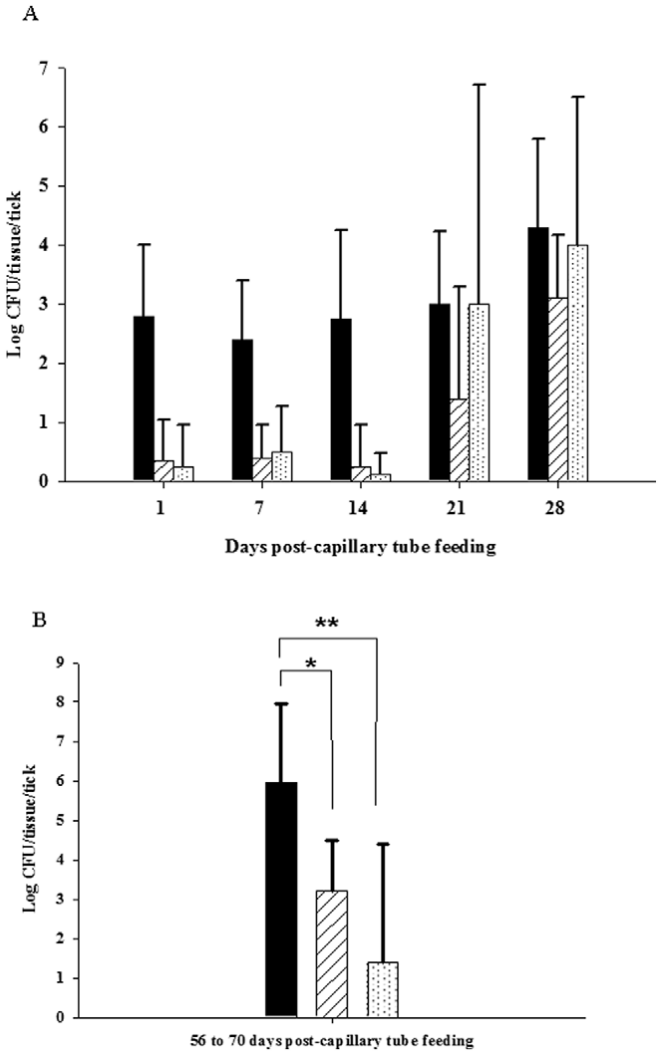
Tissue dissemination of LVS in adult *D. variabilis*. (A) Solid black bar- gut, white bar with diagonal lines- salivary gland, white bar with dots- hemolymph. For each time point the n was 5. Error bars indicate standard deviation. (B) Tissue dissemination of LVS in adult *D. variabilis* between 56 and 70 days post-capillary tube feeding. Solid black bar - gut, white bar with diagonal lines - salivary glands, white bar with dots - hemolymph. For each time point, n was 10. Error bars indicate standard deviation. _*_ Unadjusted *P* = 0.008, _**_ unadjusted *P*<0.001.

**Figure 6:**
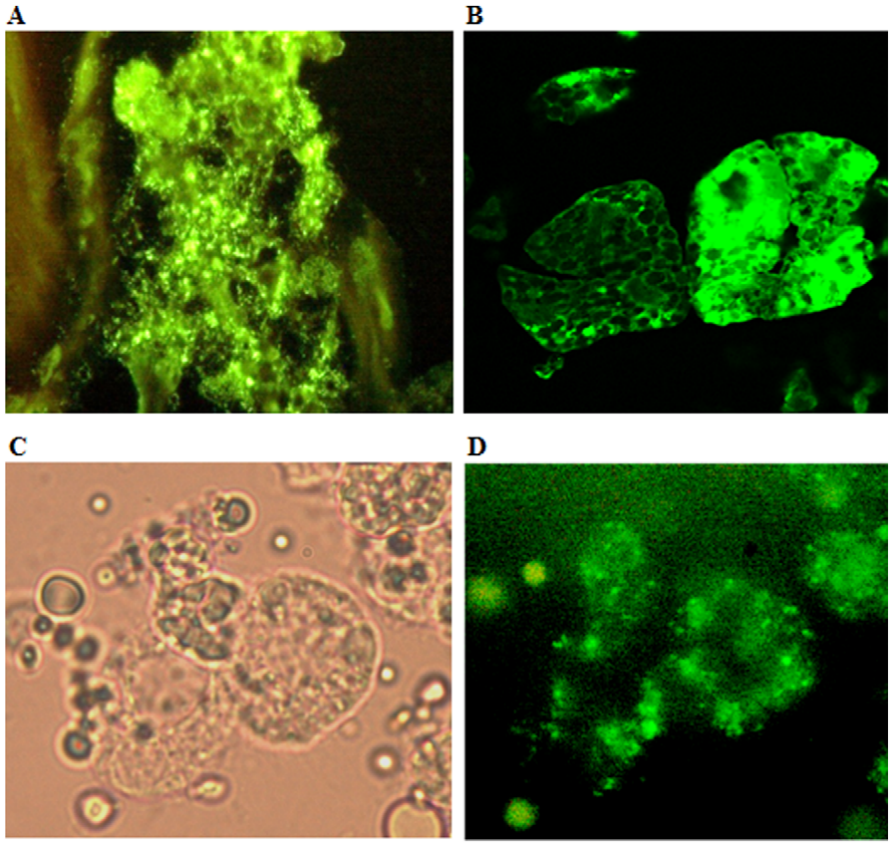
Photomicrographs of LVS colonization of tick gut, salivary glands and hemolymph. (A) Immunostained sections of adult tick gut colonized with LVS. (B) Immunostained sections of colonized adult tick salivary gland acini. (C) Overlapped image of tick hemocytes colonized with GFP expressing LVS. (D) Fluorescent image of the same tick hemocytes. (400× magnification).

To determine the dissemination of *F. tularensis* in tick tissues following colonization by ingestion, sheep fed nymphs and unfed flat adult ticks were capillary fed tick meal containing 10^7^ CFU/mL LVS and subsequently dissected on various days post-capillary feeding to determine the tissue dissemination of LVS. For adult *D. variabilis*, the primary site of LVS colonization was the tick gut through day 14 post-capillary tube feeding. By day 21 post-capillary tube feeding, LVS colonization extended to the hemolymph and salivary glands (Fig. 5A). Microscopic examination of these tissues using immunohistochemical staining confirmed that gut and salivary gland were heavily colonized by LVS and that the bacterium was present in the hemolymph, specifically intracellularly in hemocytes including granulocytes and plasmatocytes (Fig. 6 A–D). Adult ticks colonized as nymphs exhibited localization primarily in gut tissue (8.9×10^5^ CFU/tick) and salivary glands (1.6×10^3^ CFU/tick) with only low levels of localization of hemolymph (1.9×10^1^ CFU/tick) at 56 to 70 days post-capillary tube feeding (Fig. 5B).

### Mouse infectivity of LVS colonized salivary gland from *D. variabilis* adults

**Figure 7:**
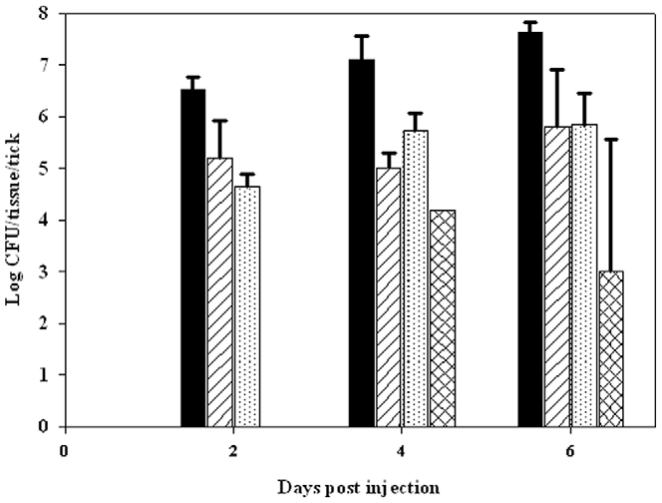
Tissue dissemination of LVS in adult *D. variabilis* post-i.h. injection. Solid black bar - gut, white bar with diagonal lines - salivary glands, white bar with dots – hemolymph and white bars with cross marks – saliva. For each time point, n was 5. LVS was detected in saliva in 0/5, 2/5, and 3/5 ticks at 2, 4, and 6 days post injection. Error bars indicate standard deviation.

To determine whether LVS disseminated to salivary glands of adult ticks are infectious, partially fed adult *D. variabilis* ticks were inoculated i.h. with LVS to achieve a rapid and consistent bacterial load in the hemolymph. Dissemination of LVS from the hemolymph into gut and the salivary gland occurred within two days post-injection (Fig. 7), and LVS was secreted into the saliva of 40% of ticks after four days post-injection with a mean level of 2.8×10^4^ CFU/µL saliva/tick and 60% of ticks with a mean level of 1.12±0.1×10^3^ CFU/µL saliva/tick after 6 days post-injection. The ID_50_ for LVS in tick salivary gland in BALB/c mice injected intra-peritoneally (i.p.) was 3 CFU as compared to 10 CFU for laboratory cultured LVS (Table 2). The mean bacterial count in liver, spleen and blood was collected from mice at their clinical end point and were 1.8×10^7^ CFU/g, 1.6×10^7^ CFU/g, and 2.8×10^5^ CFU/ml, respectively. Immunohistochemical stained sections of infected mouse liver and spleen demonstrated that liver hepatocytes and splenic cells were heavily infected by LVS.

**Table 2:** ID_50_ of LVS from *D. variabilis* salivary glands via i.p inoculation in BALB/c mice.

Group	Inoculum (CFUs of LVS)	Fraction of infected mice
A	0.05	0/6
B	0.5	1/6
C	5	4/6
D	71.3	6/6
E	493	6/6
Control	Nil*	0/5

* Control group was injected with uninfected *D. variabilis* salivary glands.

### Lack of transovarial transmission from LVS colonized gravid adult female *D. variabilis* to larvae via the egg stage

**Figure 8:**
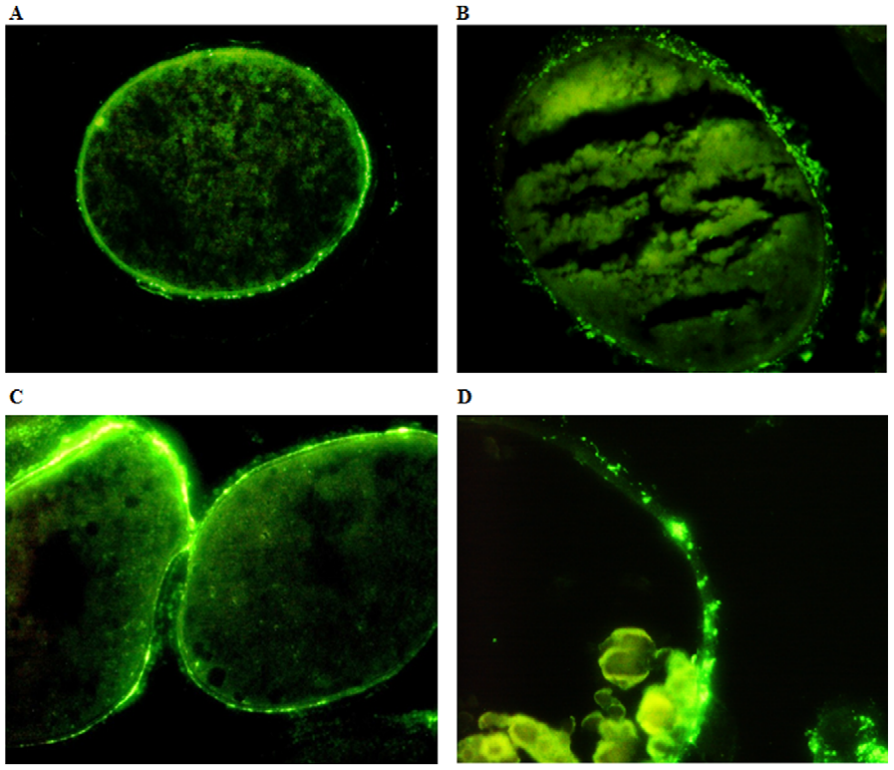
Photomicrographs of immunostained sections of colonized tick oocytes. LVS colonized the tunic propria and shell of *D. variabilis* oocytes. (A & C) 400× magnification. (B& D) 500× magnification.

To assess whether female *D. variabilis* transovarially transmit *F. tularensis* to hatched larvae, females fed on sheep to repletion were inoculated one day post-repletion with LVS i.h., and dissemination of LVS to various tissues assessed along with transovarial transmission to hatched larvae. At 14 days post-injection, the mean colonization level was 4.3×10^9^ CFU/tick and LVS was detected in hemolymph, gut, Malpighian tubules, and ovaries but only 9% of egg masses oviposited by other females in this group were positive for LVS by culture or RT-qPCR. However, when the holding temperature for the replete adult female ticks was elevated from 23°C to 27°C, the mean colonization level for the adult female ticks was 3.98×10^10^ CFU/tick and 88% of egg masses were colonized with 3.2±0.02×10^3^ CFU/egg mass. LVS was not detected in eggs deposited during the initial five days of oviposition, but ova deposited beginning approximately 7 days after initiation of oviposition were positive for LVS. Eggs hatched to larvae between 20 and 30 days post-ovipositing, but transovarial transmission of LVS to larvae was not detected by either culture or RT-qPCR in these hatched larvae (limit of detection of 10 CFU/hatched larva). The oocytes from colonized gravid females were isolated by dissection and were examined microscopically using immunohistochemistry for detection of LVS. The bacteria were demonstrated in the outer tunica propria and shell of oocytes, but not in the oocyte cytoplasm (Fig. 8 A–D). The fecundity of ticks was not affected by LVS colonization when ticks were held at 23°C as compared to non-colonized ticks. However, the mean egg mass weight of LVS colonized ticks was lower (164 mg) when compared to non-colonized ticks (320 mg) for ticks held at 27°C.

## Discussion

Eisen recently called attention to the need to more fully characterize the biology of tick vector-pathogen interactions for *F. tularensis* [11]. To date, experimental colonization of ticks by *F. tularensis* has employed acquisition feeding on infected laboratory animals; however, there are certain limitations to studies with tick colonized by this method that can be overcome by an alternative colonization method of capillary tube feeding. A significant difficulty for acquisition feeding is exposure of ticks to consistent, reproducible and known levels of bacteria. Variation in the timing of tick feeding relative to the development of bacteremia in infected laboratory animals can result in ingestion of blood with varying levels of *F. tularensis* during acquisition feeding. For tick colonization by capillary tube feeding, the number of bacteria in the inocula can be predetermined, controlled and reproduced. In addition, the amount of inocula ingested can be assessed for nymphs and adult ticks by comparing pre- and post-feeding weights. A second difficulty of acquisition feeding is the requirement for infection of laboratory animals with *F. tularensis*. These infected animals experience acute disease with a uniform fatal outcome, and therefore, additional animals must be infected whenever tick colonization experiments are done. In contrast, high numbers of ticks can be colonized by capillary tube feeding on a routine basis with minimal setup and no expenditure of live animals. A major disadvantage of capillary feeding method is that it is an artificial method in which both the agent and the vector do not experience factors associated with natural feeding. Physiological cues associated with natural tick feeding on a host may be important for tick and pathogen interactions.

For the study reported herein, replete larvae, partially fed nymphs, and unfed adults *D. variabilis* were colonized with LVS by capillary tube feeding with efficiencies of colonization of approximately 10%, 30% and 60% (day one post-capillary feeding without screening nymphs or adults for post-capillary feeding weight gain), respectively. The degree to which the various stages of *D. variabilis* were fed on uninfected animals prior to capillary tube feeding likely affected the efficiency of establishing colonization by this method for larvae and nymphs. For partially fed nymphs which gained at least 0.3 mg post-capillary feeding, the colonization efficiency was 100%, similar to that observed for acquisition fed *D. variabilis* nymphs on *F. tularensis* infected mice [21]. Colonization efficiency for larvae was less than that reported for acquisition feeding [22], likely owing to the need to use replete larvae for the capillary feeding experiments in order that these larvae would molt to nymphs.

The colonizing dose of LVS necessary to establish colonization of ticks, like the ID_50_ for mice infections, is likely extremely low based on 1.5 CFU LVS/tick being sufficient to establish colonization, albeit by the artificial route of i.h. injection. At low inocula doses, there may be a lag time for LVS to establish colonization as observed by the lowest dose for i.h. inoculation exposure requiring 14 days post-injection to be detectable. The mean quantum of LVS colonization for capillary tube fed nymphal *D. variabilis* molted to adults of 10^6^ CFU/tick is similar to that for naturally colonized adult questing *D. variabilis* [23]. Coevolution of tick vectors with *F. tularensis* may allow this bacterium to avoid killing by tick innate immune mechanisms resulting in high quanta of colonization [24], [25]. However, both naturally and capillary tube fed ticks colonized with *F. tularensis* showed a wide range of colonization quanta of 10^5^ to 10^10^ CFU/tick [21], [22]. The mechanism(s) underlying this variability in colonization level within a population of similarly exposed ticks is not apparent.

Previously reported transstadial transmission studies of *F. tularensis* in *D. variabilis* ticks have been by acquisition feeding on *F. tularensis* infected lab animals [15], [22]. The current study demonstrates that transstadial transmission occurs in ticks colonized by capillary tube feeding. Only minimal mortality was observed for capillary tube fed *D. variabilis* colonized with LVS for larvae molted to nymphs and nymphs molted to adults with no statistical difference observed for ticks fed tick meal with or without LVS. However, Reese *et al.* reported a high fitness cost associated with *F. tularensis* subspecies *tularensis* colonization of *D. variabilis* larvae molted to nymphs, but not for nymphs molted to adults [21], [22]. This difference may be related to the attenuated nature of LVS compared to *F. tularensis* subspecies *tularensis* strains or factors originating from animal passage versus laboratory growth of LVS.

Francis (1927) described the qualitative microscopic changes in *D. andersoni* ticks following *F. tularensis* colonization. The current study reports the systematic quantification of *F. tularensis* colonization of various tick tissues. Following ingestion, we observed that LVS required >14 days to multiply in the gut before dissemination to the hemocoel and other tissues. Once disseminated into the hemocoel, LVS readily and heavily infected hemocytes. Other tissues including Malpighian tubules, ovaries and salivary glands were also colonized. Colonization of the hemolymph may be cleared at longer duration of colonization as reflected by restriction of colonization to the gut in adults colonized as nymphs. In contrast to our finding, Francis did not observed *F. tularensis* dissemination to ovaries and salivary glands [26]. Although *F. tularensis* is transmitted via tick bites, the direct detection of *F. tularensis* in the tick saliva has only recently been reported [27]. The chemically-induced salivary secretion technique reported herein to demonstrate LVS in salivary secretions of *D. variabilis* was used previously by others to determine physiological components of tick saliva and to detect other tick borne pathogens [28], [29], [30], [31]. The virulence of LVS recovered from the tick salivary gland was also determined by determining the ID_50_ which was found to be the same order of magnitude as that for laboratory cultured LVS. Although the ID_50_ was similar, others have evidence that components of tick saliva may increase the virulence of various tick pathogens including *F. tularensis* by suppressing host immune response [32], [33], [34].

Transovarial transmission of *F. tularensis* in *D. variabilis* ticks was first reported in 1936, but later experiments could not reproduce these results [15], [17]. For our experiment, adult female ticks were inoculated with LVS i.h. within one day of feeding to repletion on sheep, LVS was detected in ova oviposited on day eleven (seven days after beginning of oviposition); however, we found no evidence of transovarial transmission of LVS to hatched larvae. We observed transmission of LVS to the tick oocyte shell and tunica propria, but not to the cytoplasm of the oocyte. The observation that LVS does not penetrate into the oocyte cytoplasm may explain the lack of transovarial transmission to larvae. RT- PCR did not detect LVS DNA in hatched larvae confirming the larvae were free of LVS and not just free of viable but non-culturable LVS. The failure of transmission of LVS to hatch larvae could be due to the time required for dissemination of the inoculation from the hemocoel to the ovaries or the developmental stage of the ova at the time of *F. tularensis* colonization of the reproductive tract. Because oogonia originate during the larval stage and develop to oocytes in the nymphal stage [35], it is possible that for transovarial transmission to occur that colonization of oogonia or oocytes by *F. tularensis* must occur during larval or nymph feeding on infected host. In addition, the level of LVS colonization of ova may reduce hatching as observed for one egg clutch with 10^7^ CFU/100 ova which failed to produce any larvae. This phenomenon is also seen in *Borrelia* infected ixodid ticks [36].

The overwintering reservoir from which *F. tularensis* initials seasonal epizootics is not known, although overwintering ticks have been proposed as a possible reservoir [11]. The overwintering period for ticks varies with latitude, but in eastern and central United States its duration is 5 to 6 months [37], [38], [39]. Both larval and adult *D. variabilis* overwinter in a natural environment similar to the tularemia endemic region [40]. We observed that LVS colonization persisted in capillary tube fed nymphs molted to adults and unfed adults for 4 and 5.5 months, respectively, which were the longest time points in these experiments. These results are compatible with adult *D. variabilis* ticks maintenance of colonization through the winter period as an inter-epizootic reservoir for *F. tularensis* in its enzootic area. Whether unfed colonized larvae may serve as an overwintering reservoir for *F. tularensis* must await clarification of whether transovarial transmission of *F. tularensis* can occur in *D. variabilis*.

## Materials and Methods

### Ticks, bacterial strain and growth conditions

*D. variabilis* larvae, nymphs, and adults were obtained from the Tick Rearing Facility, Department of Entomology and Plant Pathology, Oklahoma State University (Stillwater, OK). Larvae were collected after they were fed to repletion on rabbits. Nymphs were partially fed on sheep until they weighed approximate ≥4.5 mg/nymph. Unfed flat adult ticks were used for capillary tube feeding studies. Adult ticks used for salivary induction experiments were partially fed on sheep for five to six days. Female ticks used to study transovarial transmission were fed to repletion on sheep. Capillary tube feeding of nymphs and adult ticks was initially assessed by weighing ticks before and after feeding. Only those ticks (nymphs/flat adults) that gained ≥0.3 mg after capillary tube feeding were used for the experiment unless otherwise specified.

*F. tularensis* subsp. *holarctica* strain LVS was supplied by the Oklahoma State Department of Health. Green fluorescent protein (GFP) expressing plasmid (pFNLTP6 *gro-gfp*) was a gift of Thomas C. Zhart (Medical College Wisconsin, Milwaukee, Wisconsin), and electroporated into LVS [41]. LVS was grown on chocolate agar plates (Hardy diagnostics, Santa Monica, CA.) at 37°C in 5% CO_2_ for 72 h. The BBL Prompt Inoculation System (BD Diagnostics, Franklin Lakes, NJ) was used to prepare inocula for colonization of LVS. All chemicals used in the study were purchased from Sigma (St Louise, MO) unless indicated otherwise.

### Capillary tube feeding of larvae, nymphs and adult ticks

The ticks were surface disinfected by washing in 30% hydrogen peroxide, distilled water and 70% isopropyl alcohol for 5 seconds each. After the washing, adult ticks were immobilized dorsal side up on adhesive part of duct tape placed over double-sided adhesive tape (3M Scotch brand, St Paul, Minnesota) in a 100 by 15 mm Petri dish base. Ticks were then further immobilized by applying single-sided adhesive tape over 1/4^th^ of the caudal region. Larvae and nymphs were immobilized with their dorsal side down on double-sided adhesive tape on a Petri dish base. For capillary tube feeding, 10 µL (internal diameter of 0.55 mm), 9 µL (internal diameter of 0.48 mm) and 35 µL (internal diameter of 0.79 mm) glass capillary tubes (Drummond Scientific Company, Broomall, PA) were used for larvae, nymphs and the adult ticks, respectively. The ends of the tubes were positioned over the hypostome of the tick while the other end rested on the edge of the Petri dish taped with a double-sided adhesive tape [19]. Feeding medium for larvae, nymphs, and adult ticks was Minimum Essential Media (MEM) (GIBCO Grand Island, NY) with 10% fetal bovine serum (Hyclone, Logan, UT.). The feeding medium was spiked with *F. tularensis* subsp. *holarctica* strain LVS at 10^7^ CFU/ml. The tick meal was then introduced into the capillary tubes, and larvae and adults were fed for 18 hours at 30°C and 90% relative humidity. The nymphs were fed either for 6 or 18 hours under the same conditions. The change in duration of nymphal feeding did not change either the percent of nymphs gaining >0.3 mg post-capillary feeding or the mean CFU/nymph of LVS at 1 day post-capillary feeding. After feeding ticks were either surface disinfected by washing in 30% hydrogen peroxide, distilled water and 70% isopropyl alcohol for 5 seconds and minced for determination of CFUs or were maintained in microcentrifuge tubes capped with moistened cotton plugs and kept in a humidity chamber (relative humidity of >90%) at 23°C (unless specified otherwise) with automated artificial lighting to simulate a 12 h day-night cycle. Determination of CFU per tick was done at one day post-capillary feeding and then at an interval of 7 days. To determine the bacterial numbers in ticks at various times of colonization, individual ticks were minced with a scalpel blade, bacteria extracted by incubation in PBS containing 64 µg/mL ampicillin for 2 h at room temperature on a rotor platform mixer (Boekel Scientific, Feasterville, PA.), serially diluted in PBS containing 64 µg/mL ampicillin, and plated on chocolate agar plates for CFU determination following incubation at 37°C in 5% CO_2_ for 72 h. To determine the bacterial numbers in tissues at varying periods of post-capillary tube feeding, individual ticks were dissected into gut, salivary glands, and ovaries under sterile conditions using a dissecting microscope. Hemolymph was collected into sterile glass capillary tubes from the cut ends of a tick leg. The tick tissues were placed in PBS containing 64 µg/mL ampicillin and processed as described above for whole ticks.

### Immunohistochemistry

For immunohistochemical analysis, ticks were cut longitudinally in half and ovaries from gravid females were fixed in Carson's fixative, embedded with paraffin and sectioned and affixed to glass slides. After deparaffinizing, the sections were incubated with phosphate buffered saline with 0.05% Tween 20 (PBST) at RT for 15 min. and then incubated at 37°C for 1 h with *F. tularensis* antiserum (Beckton Dickinson, Sparks, Maryland) at 1/60 dilution in PBST. Adsorbed antiserum was used as negative control. After washing the slides with PBST five times followed by a final washing with distilled water, the sections were incubated with FITC conjugated secondary antibody (KPL, Gaithersburg, Maryland) at 1/60 dilution in PBST at 37°C for 30 min. The sections were then washed in PBST twice, PBS once and finally washed with distilled water. The slides were dried and visualized using Nikon Eclipse 50i epi-fluorescence microscope and Nikon digital sight DS-5M-L1 digital camera. For visualizing LVS in tick hemolymph, ticks were capillary tube fed with GFP expressing LVS and after 1, 2, 3, and 4 weeks post-capillary tube feeding; hemolymph was collected and placed directly on glass slide with coverslip, and visualized using the epi-fluorescent microscope.

### Real-time quantitative PCR

Real- time quantitative PCR was done to determine whether the adult ticks that cleared LVS infection after capillary feeding had viable but non-culturable bacteria and also to determine whether LVS had been transovarially transmitted to hatched larvae. For RT-qPCR reactions, a 97 bp product of *F. tularensis* insertion sequence-2 was amplified with the primers ISFtu2F and ISFtu2R [42]. Each sample was analyzed using Fast SYBR green master mix on an AB 7500 Fast Real-Time PCR System (Applied Biosystems, Foster City, CA.). During each analysis a negative control (no template) was processed and the amplification product was confirmed by analyzing the dissociation curve. RT-PCR reaction (20 µL) - 10 µL Fast SYBR green master mix, 6 µl DNase RNase-free water, 1 µL Forward primer (ISFtu2F), 1 µL Reverse primer(ISFtu2R) and 2 µL template. Cycling conditions were 95°C for 20 seconds, followed by 34 cycles of 95°C for 10 seconds and 60°C for 30 seconds. Genome equivalents were calculated based on standard curve obtained by plotting cycle threshold value and different concentrations of LVS DNA. The final value for each sample is calculated by multiplying with the dilution factor. The sample used for PCR was total DNA from tick (tick minceate in 100 µl PBS) extracted using DNeasy Tissue Kit (Qiagen, Valencia, CA.), with a final elution volume of 50 µL.

### Intra-hemocoelic inoculation and salivary induction in ticks

To determine the lowest colonization dose for ticks and to colonize gravid females and partially fed adult ticks, 1 µL of the inoculum containing 10^8^ CFU/mL of *F. tularensis* subsp. *holarctica* strain LVS in PBS was injected i.h. in the ventral region of the tick, medial to the caudal most coxa using 10 µL custom made Hamilton syringe with a 0.5 inch, 33 gauge needle (Hamilton Company, Reno, NV.). Injection of gravid females was done in the left or right spiracles. For the detection of LVS in tick saliva, partially fed adult ticks were injected with LVS via i.h. route, and the ticks were held in humidity chamber at 27°C. For collecting saliva, partially fed LVS-colonized adult ticks were immobilized dorsal side up on the sticky part of duct tape placed on a double-sided adhesive tape. Ticks were then injected i.h. with 4 µL of 1 mM dopamine, 1 mM theophilline and 3% DMSO in PBS (pH 7.3) [31] every 15 min. for 1 h. Saliva was collected in a 10 µL (internal diameter of 0.0219 inch) volume glass capillary tubes (Drummond Scientific Company, Broomall, PA) placed over the hypostome of the tick. The capillary tube for collecting saliva was held in place using modeling clay.

### Infective dose 50 in BALB/c mice

The Animal Care and Use Protocol for mouse ID_50_ experiments was approved by the Oklahoma State University Institutional Animal Care and Use Committee (IACUC protocol VM-10-[1030410]). To determine infectivity of LVS recovered from ticks, salivary glands from four partially fed adult ticks (colonized with LVS four days previously via i.h. route and held at 28°C) were dissected under sterile conditions and minced in 200 µL PBS containing 64 µg/mL ampicillin. The salivary glands in PBS-ampicillin were diluted to make appropriate inoculum size. Five experimental groups of BALB/c mice (six mice in each group) were injected i.p. with 0.05 CFU, 0.5 CFU, 5 CFU, 71.3 CFU, and 493 CFU, respectively. Another five experimental groups of BALB/c mice (six mice in each group) were injected i.p. with 0.38 CFU, 3.8 CFU, 38 CFU, 193 CFU and 387 CFU of laboratory cultured LVS respectively One control group of five mice were injected with salivary glands in PBS-ampicillin from uninfected ticks (injected with PBS four days previously via i.h. route and held at 28°C). All the mice that showed the clinical endpoint (ruffled haircoat, huddling, lethargy, and decreased mobility) were euthanized. Liver and spleen were aseptically removed form the mice, weighed and homogenized. Blood was collected from the heart immediately after euthanizing the mice and serial 10 fold dilutions were made and plated on chocolate agar plates and CFUs were counted after 72 h of incubation at 37°C and 5% CO_2_. The data from the experiment were used to calculate ID 50 using Reed-Muench method [43].

### Statistical analysis

LVS colonization in different groups of *D. variabilis* ticks during adult colonization, transstadial transmission from larva to nymphs, and nymph to adult were compared by using 1-way analysis of variance on log-transformed data followed by pairwise multiple comparison of mean CFU values using Holm-Sidak tests. Overall significance level for Holm-Sidak tests was *P* = 0.05. The same method was also used to compare LVS tissue colonization of two months post-capillary tube fed-adult ticks. Mann-Whitney Rank Sum test was performed to determine the statistical difference in the mean CFU/colonized-tick between molted adult male and female *D. variabilis*. All statistical analyses were performed with SigmaPlot v11.0 software package (Systat Software Inc., Chicago, IL).
